# Supplementary material for: Glucose represses dendritic cell-induced T cell responses
Source: Nat Commun. 2017 May 30;8:15620. doi: 10.1038/ncomms15620 (PMC5459989; doi:10.1038/ncomms15620)
Supplement: Supplementary Information — Supplementary Figures and Supplementary Table [file ncomms15620-s1.pdf]

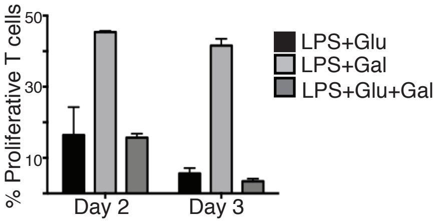

Supplementary Figure 1.
GM-DCs were pulsed with SIINFEKL peptide for 8 hours +/- LPS (100 ng/ml), washed and placed in media containing either 10 mM glucose (Glc), 10mM galactose (Gal) or 10 mM glucose plus 10mM galactose (Glc+Gal). GM-DCs were then maintained in these culture conditions for 2 or 3 days. On the indicated day post LPS stimulation GM-DCs were washed and normal media containing 10 mM glucose was added before the addition of purified CFSE labelled OT-I T cells. After a co-culture period of 48 hours the OT-I T cells were analysed by flow cytometry for proliferation as measured by CFSE dilution. Data is mean +/- range of two separate experiments.

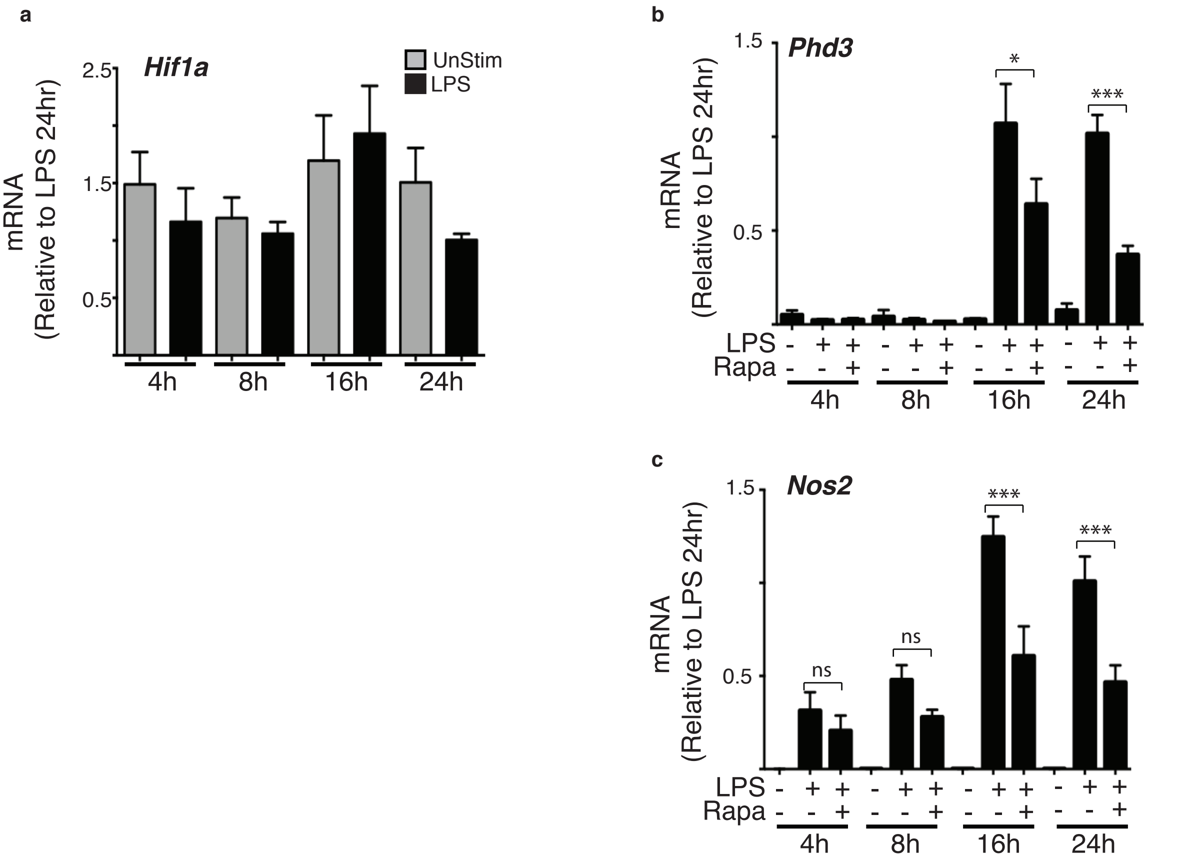

Supplementary Figure 2.
GM-DC were left unstimulated (UnStim) or stimulated with LPS (100 ng/ml) in the presence or absence of rapamycin (20 nM) (as indicated) for 4, 6, 16 and 24 hours, then analysed by qPCR for the expression of (a) *Hif1a* (b) *Phd3* or (c) *Nos2* mRNA. Data is mean +/- SEM of three experiments. Data was analysed using a 1-way ANOVA with a Tukey post-test (*p<0.05, ***p<0.001, ns = not significant)

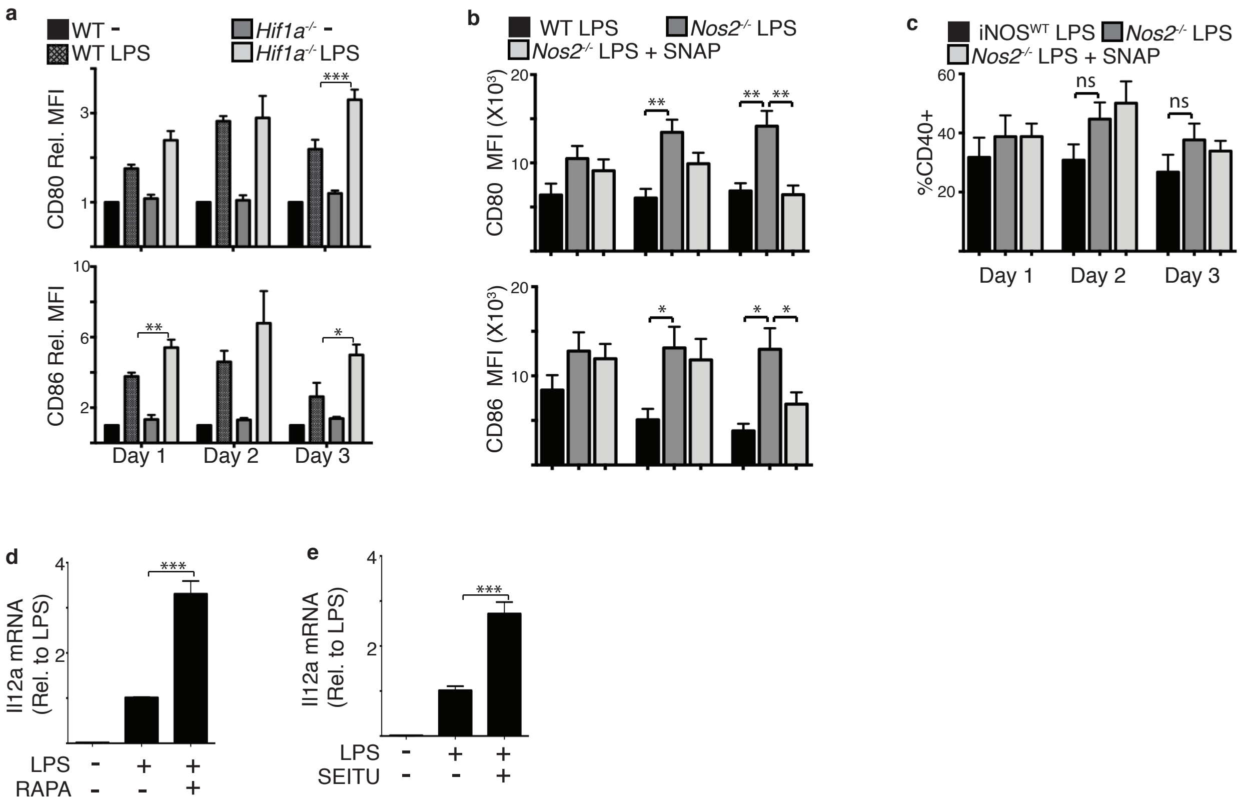

Supplementary Figure 3.
(a) HIF-1α$^{flox/flox}$ (WT) or HIF-1α$^{flox/flox}$ VavCre (*Hif1a*$^{-/-}$) GM-DCs were pulsed with SIINFEKL peptide +/- LPS (100 ng/ml) for 1, 2 or 3 days. CD80 and CD86 expression (MFI, mean fluorescent intensity) was analysed by flow cytometry. **(b,c)** WT and iNOS-null (*Nos2*$^{-/-}$) GM-DCs were pulsed with SIINFEKL peptide +/- LPS (100 ng/ml) for 1, 2 or 3 days. GM-DCs were treated with SNAP (500μM) every 5 hours, where indicated, and CD80 and CD86 (b) and CD40 (c) expression was analysed by flow cytometry. (d,e) GM-DCs were left unstimulated or stimulated for 20 hours in the presence or absence of (d) rapamycin (20 nM) or (e) SEITU (500 μM) and *IL12a* mRNA was analysed by qPCR. Data is mean +/- SEM of three (d,e), six (a) or seven (b,c) separate cultures. Data was analysed using a 1-way ANOVA with a Tukey post-test (*$p<0.05$, ** $p<0.01$, ***$p<0.001$)

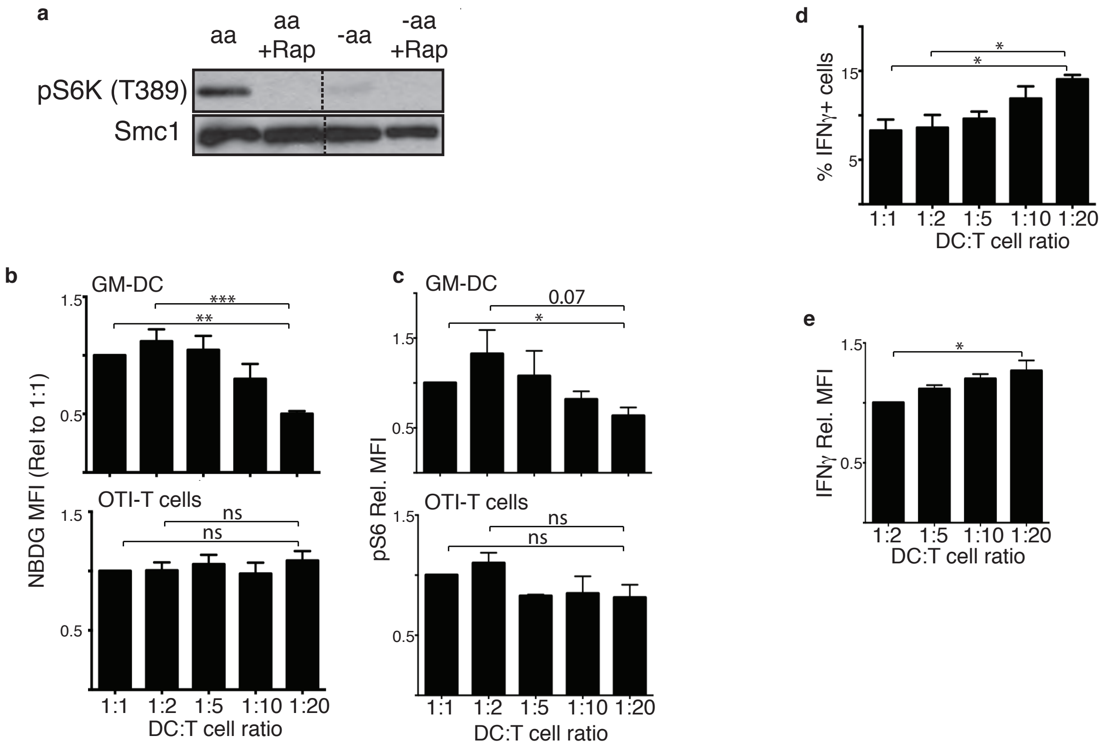

Supplementary Figure 4.
(a) GM-DC were left in complete media +/- rapamycin (20 nM) or deprived of amino acids for 1 hour +/- rapamycin and analysed by immunoblot analysis for the levels of phosphorylated p70 S6-kinase on thronine 389 (pS6K-T389). Structural Maintenance Of Chromosomes protein (Smc1) expression was assessed as a loading control. **(b-e)** GM-DCs were pulsed with SIINFEKL peptide for 6 h +/- LPS (100 ng/ml), washed and purified OT-I T cells were added at different T:DC ratios for 18 h. **(b)** NBDG uptake and **(c)** pS6 levels in GM-DC (top) and CD8 T cells (bottom) was measured by flow cytometry. **(d,e)** IFNγ production was assessed in CD8 T cells by intracellular flow cytometry; % IFNγ positive T cells (d) and the mean fluorescent intensity (MFI) of IFNγ expression in IFNγ positive T cells (e) is shown. Data is representative of two separate experiments (a) and mean +/- SEM five separate experiments (b-e). Data was analysed using a 1-way ANOVA with a Tukey post-test (*p<0.05, ** p<0.01, ***p<0.001).

a

Gating Scheme for T cell analysis

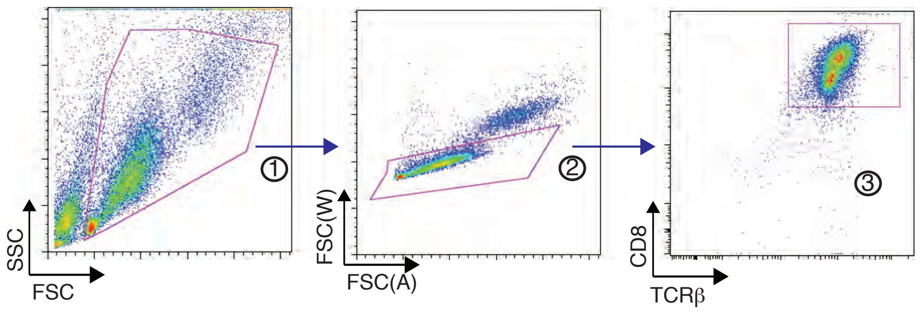

1. Cell gate
2. Doublet discrimination
3. TCRβ+ CD8+ OTI T cells

b

Gating Scheme for GM-DC analysis

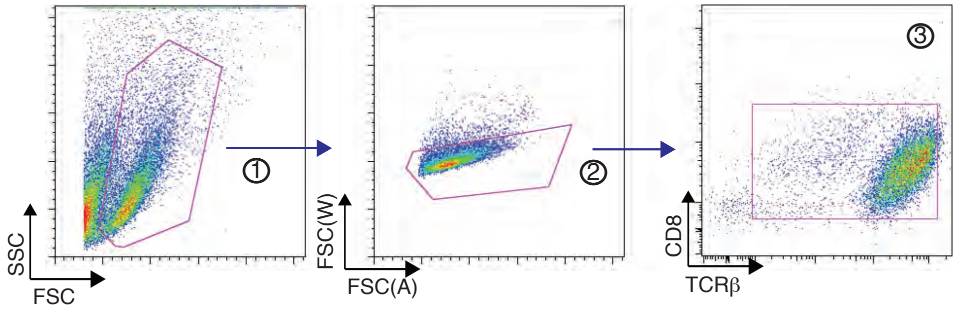

1. Cell gate
2. Doublet discrimination
3. CD11c+ MHCII+ GM-DC

Supplementary Figure 5.
(a) Gating stragegy for the analysis of OTI CD8 T cells in Fig.2, Fig. 7i-l, Fig. 8h-j, Supplementary Figure 1 and Supplementary Figure 4d, (b) Gating stragegy for the analysis of GM-DC in Fig. 1b-d, Fig. 7a-d and Supplementary Figure 3a-c

a

Gating Scheme for *in vivo* experiments

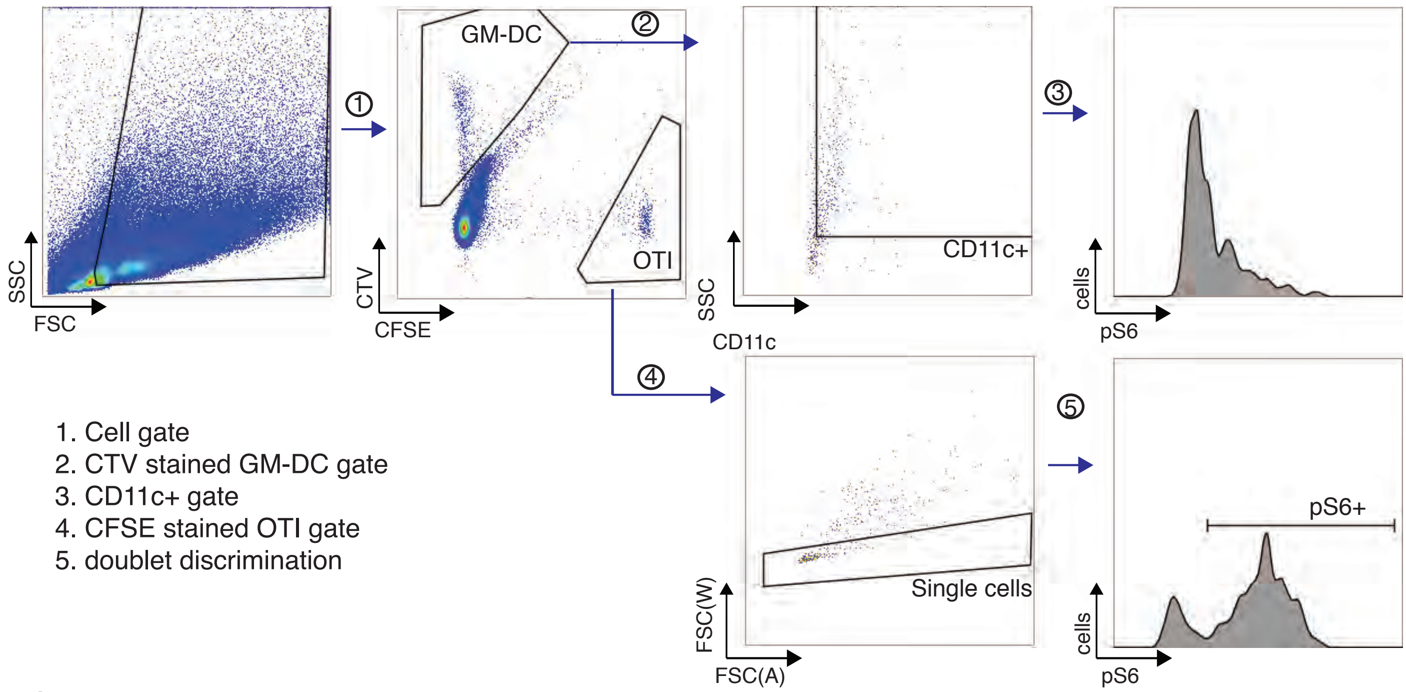

b

Gating Scheme for DC/T cell co-cultures

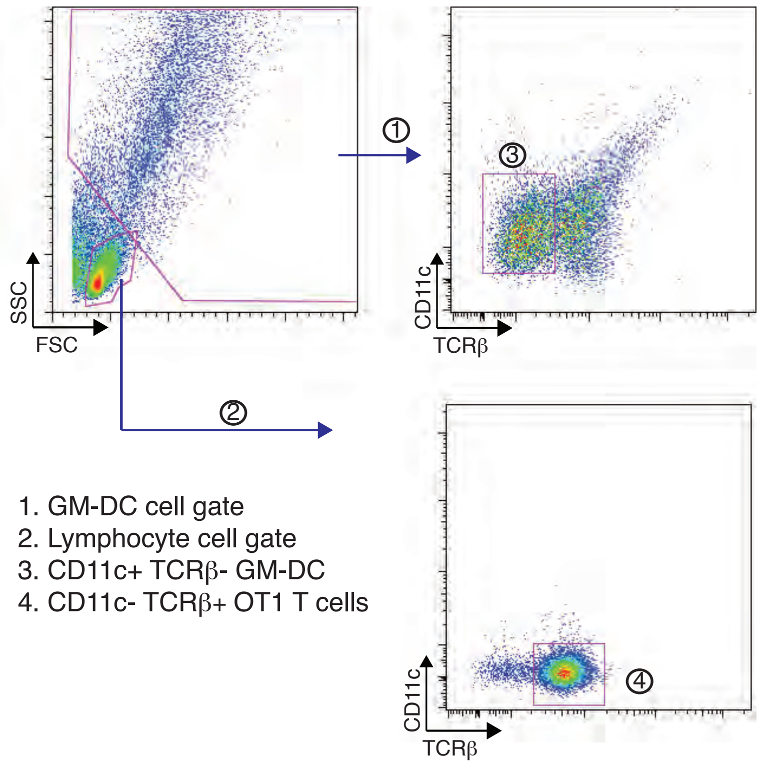

Supplementary Figure 6.
(a) Gating stragegy for the analysis of adoptively transfered GM-DC and OTI CD8 T cells in Fig.8k-m. (b) Gating stragegy for the analysis of GM-DC and OTI CD8 T cells in co-culture experiments in Fig.8b-c and Supplementary Figure 4b-c.

Supplementary Figure 7

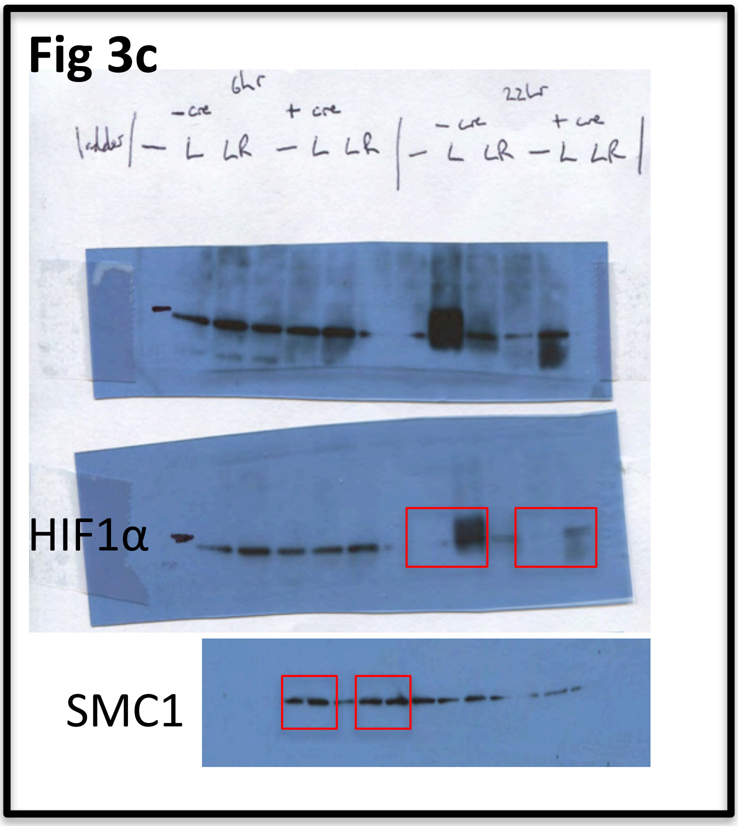

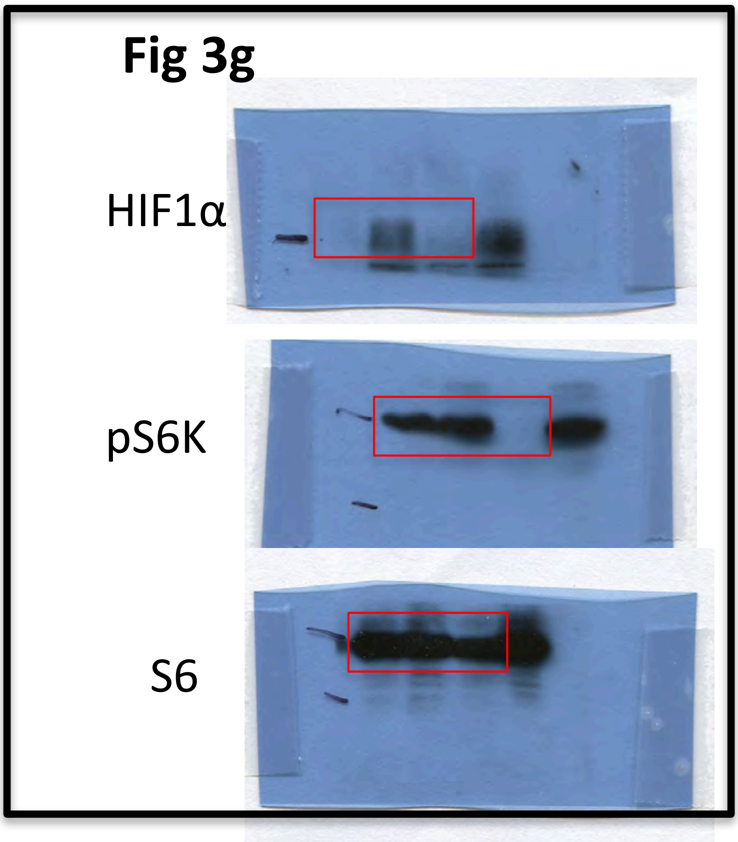

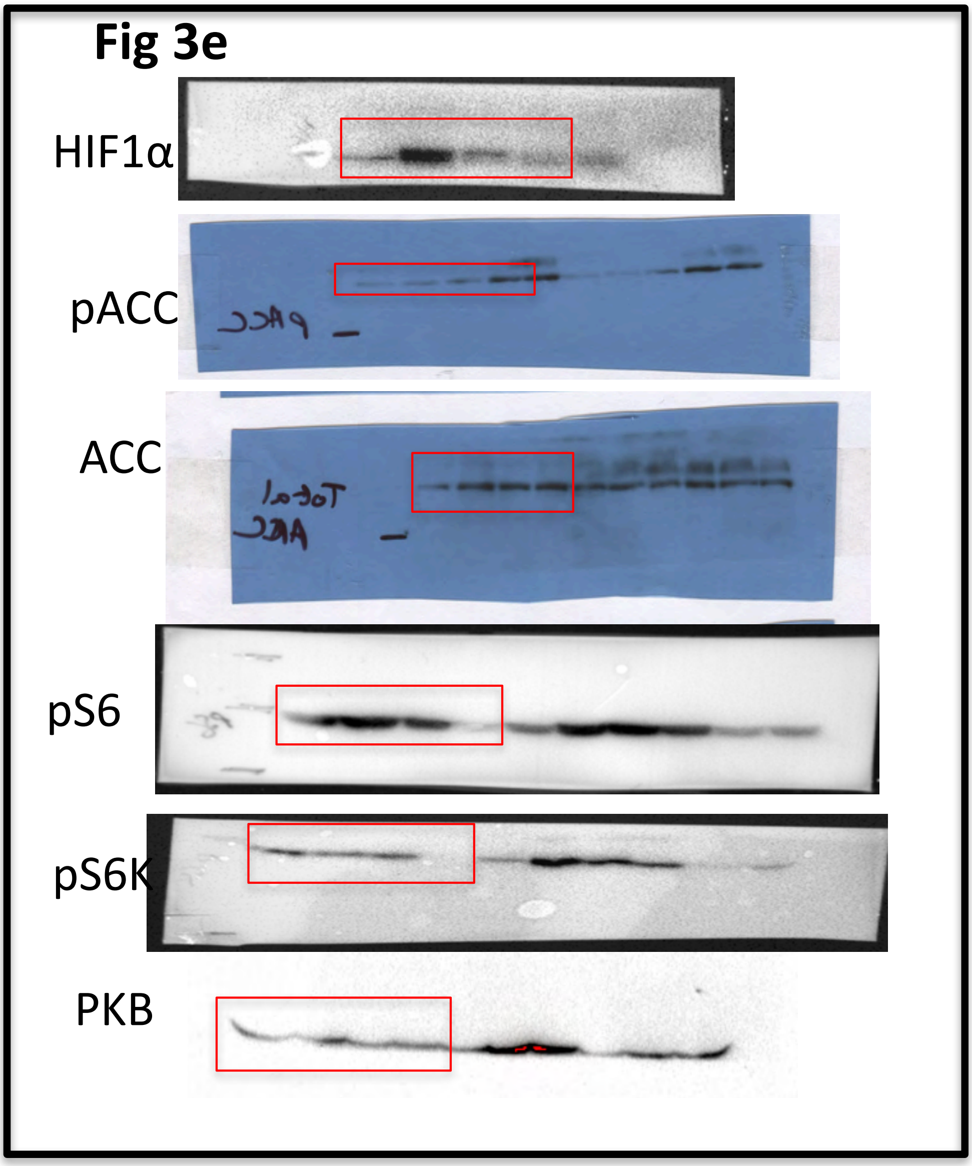

Supplementary Figure 7

Fig 5c

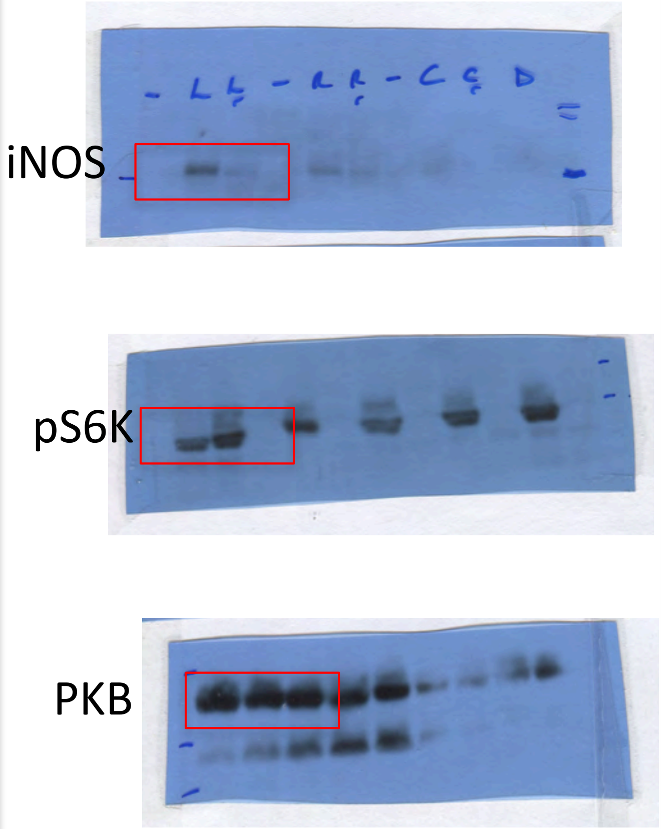

Fig 5h

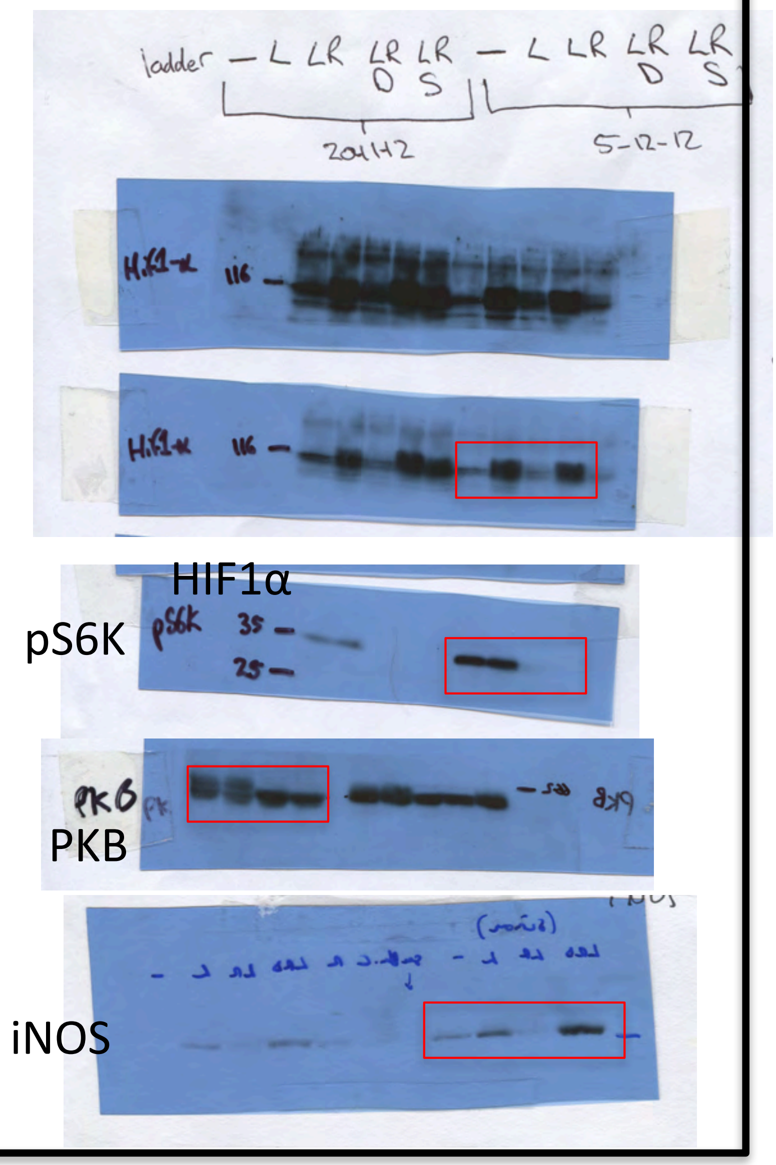

Fig 5e

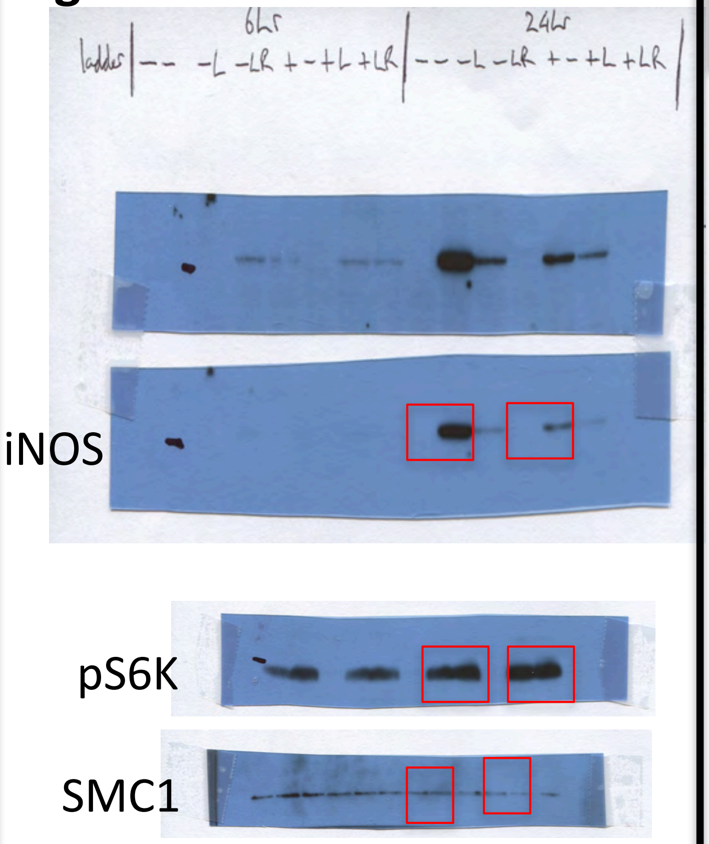

Supplementary Figure 7

Fig 6a

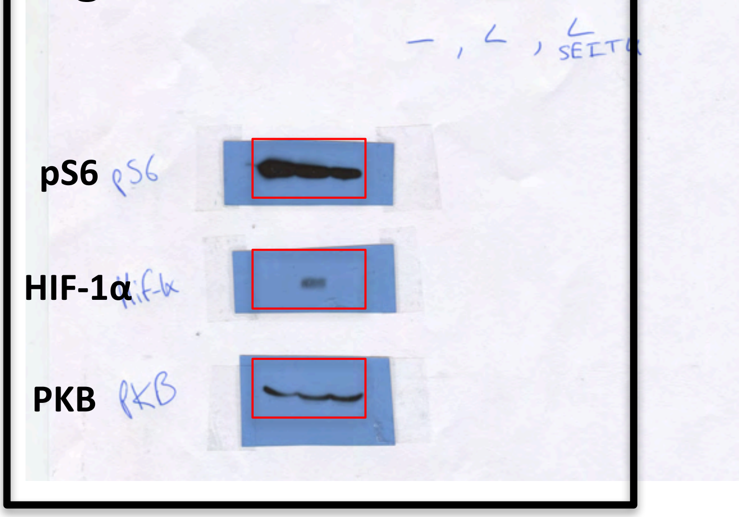

Fig 6f

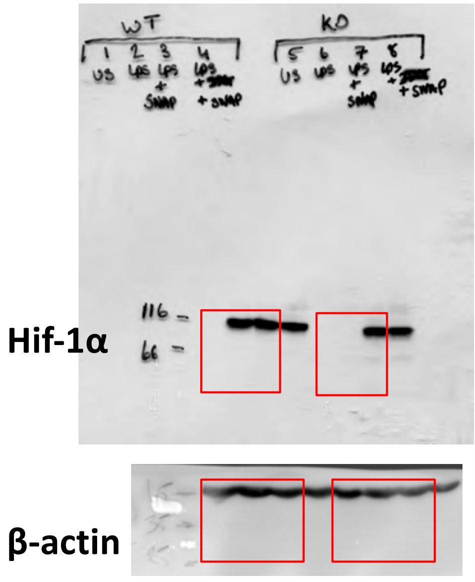

Fig 6c

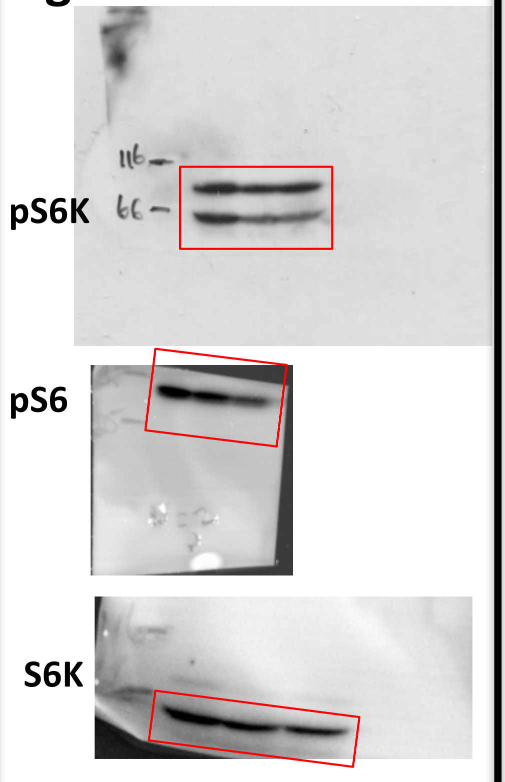

Fig 6g

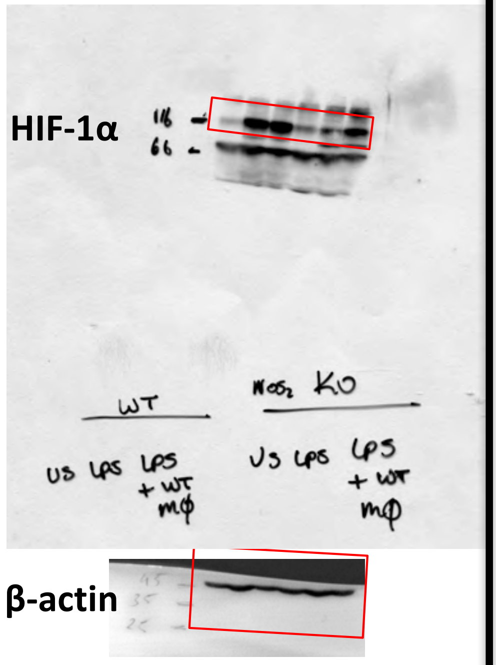

Fig 6h

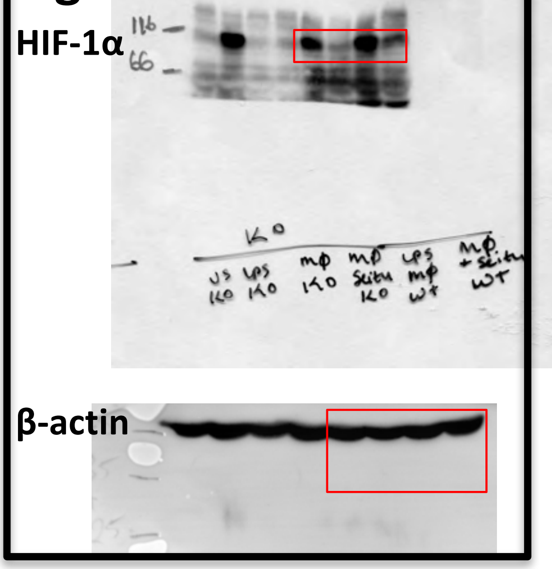

Supplementary Figure 7

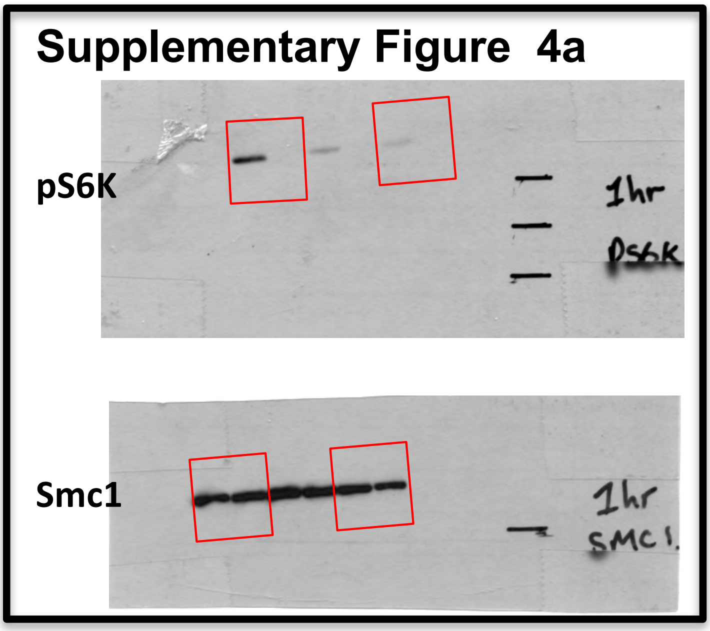

Supplementary Table 1:

RTPCR primers

**Gene**	**Forward sequence 5’-3’**	**Reverse sequence 5’-3’**
Phd3	CTGGTCCTGTACTGCGGGAG	GACCCCTCCGTGTAACTTGG
Nos2	GTCAACTGCAAGAGAACGGAGAAC	CTGCGGGGAGCCATTTTG
Pkf	GCGGCGTGTGTTCATTGTAG	CACTTCTCGTTCCGGAGCAC
Pkm2	GCTATTCGAGGAACTCCGCC	AAGGTACAGGCACTACACGC
Slc2a1	GGAATCGTCGTTGGCATCCT	CGAAGCTTCTTCAGCACACTC
Il12a	AGTCCCGAAACCTGCTGAAG	GCTCCCTCTTGTTGTGGAAGA
Rplp0	CATGTCGCTCCGAGGGAAG	CAGCAGCTGGCACCTTATTG
Il10	GGACAACATACTGCTAACCGAC	AGAAATCGATGACAGCGCCTC
TNF	AGG CAC TCC CCC AAA AGA TG	TGG TTT GTG AGT GTG AGG GTC
